# Delayed diagnosis of nail unit squamous cell carcinoma associated with chronic lichen planus like graft-versus-host disease: A diagnostic lesson

**DOI:** 10.1016/j.jdcr.2026.08.010

**Published:** 2026-08-17

**Authors:** Kevin Chen, Roberto Novoa, Ryanne Brown, Nour Kibbi, Jasmine Rana

**Affiliations:** a Stanford Department of Dermatology, Redwood City, California; b Stanford Department of Pathology, Palo Alto, California

**Keywords:** bone marrow transplantation, chronic graft-versus-host disease, dermatologic oncology, graft-versus-host disease, human papillomavirus, immunosuppression, Mohs micrographic surgery, nail dystrophy, nail unit squamous cell carcinoma, subungual malignancy

## Introduction

Squamous cell carcinoma (SCC) is an increasingly recognized complication among bone marrow transplantation (BMT) recipients with chronic graft-versus-host disease (cGVHD).^1^ cGVHD affects up to half of BMT survivors and is an established independent risk factor for SCC, particularly at mucocutaneous sites.^2^ Nail unit SCC is rare, and when it occurs in immunocompromised patients it is frequently associated with high-risk human papillomavirus (HPV).^3^ As chronic nail dystrophy, inflammation, and pain are common in cGVHD, early malignant change may be clinically obscured, creating potential for delayed diagnosis. We report a case of subungual SCC arising amid longstanding nail cGVHD highlighting the importance of clinical vigilance for focal nail changes and a low threshold for biopsy when 1 nail behaves differently from the rest.

### Report of a case

A man in his 50s with acute monocytic leukemia underwent allogeneic BMT 25 years prior. Six months post-transplant, he developed cGVHD involving the oral mucosa, liver, lungs, eyes, skin, and nails, treated with sirolimus, corticosteroids, and mycophenolate mofetil. Although cGVHD resolved at other sites, nail changes including pterygium formation and lichen planus-like alterations persisted for over 2 decades (Fig 1). This prolonged course created a background of chronic nail dystrophy that could reasonably be attributed to cGVHD alone, thereby complicating recognition of superimposed pathology.

**Fig 1:**
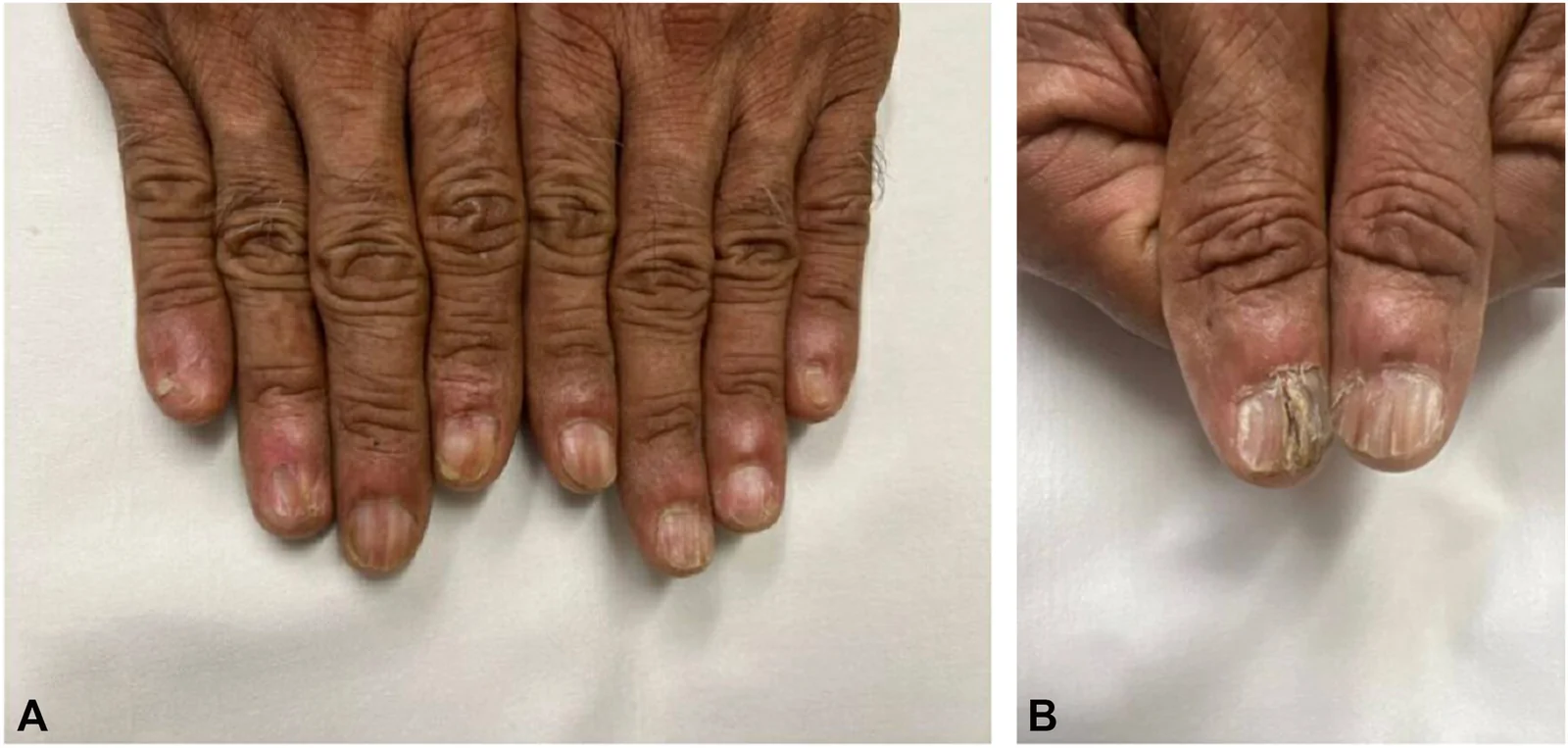
Clinical images of fingernails. **A,** Pterygium formation and lichen planus-like presentation consistent with nail cGVHD across multiple digits. **B,** Right first fingernail with longitudinal splitting, tenderness, and bleeding, the outlier nail prompting biopsy.

At 22 years post-transplant and off immunosuppression, he presented with persistent bleeding, tenderness, and longitudinal splitting isolated to the right first fingernail (R1F) (Fig 1). Over the preceding 2 years, this nail had been treated with intralesional triamcinolone injections to the nail matrix without improvement. Critically, while all 20 nails exhibited chronic cGVHD changes, the R1F showed focal new symptoms and non-response to therapy, distinguishing it from the remaining nails and raising concern for superimposed malignancy. The unilateral nature of these changes, coupled with progressive symptoms despite directed treatment, was particularly concerning and suggested that this nail was following a clinical course distinct from the patient’s baseline cGVHD-associated dystrophy. There was no history of local trauma to the affected digit, and the patient had no personal or family history of SCC or other malignancies.

Biopsy of the nail bed and distal matrix of the R1F revealed SCC in situ. Although p16 expression was wild-type and in situ hybridization for high-risk HPV subtypes was negative, verrucous architecture and cytologic features raised suspicion for possible HPV-mediated oncogenesis involving a low-risk or untyped subtype (Fig 2). The patient underwent Mohs micrographic surgery with no deeper invasion and remains in clinical remission at 2 years.

**Fig 2:**
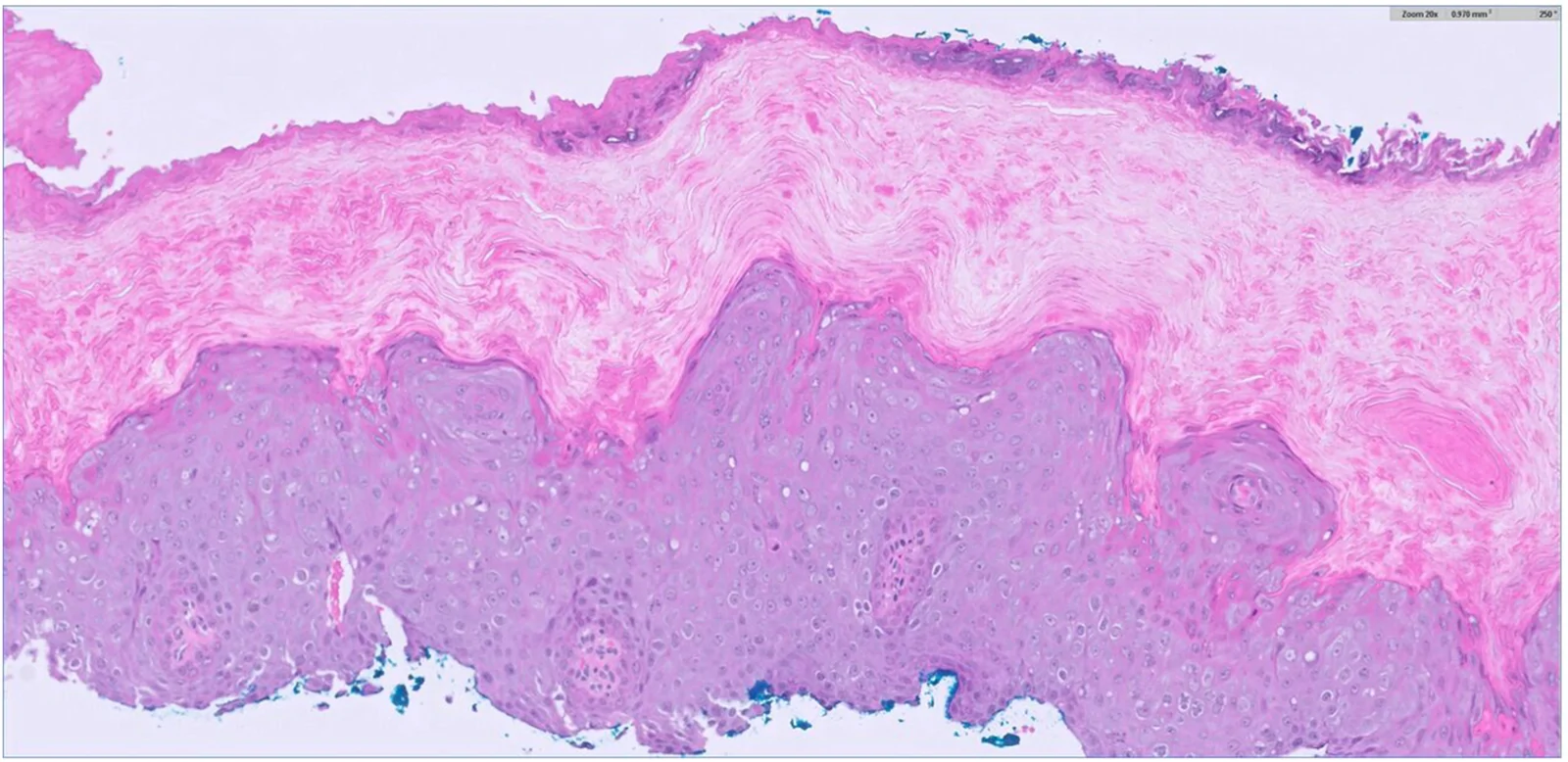
Histopathologic features of subungual SCC in situ. The nail bed demonstrates verrucous hyperplasia with full-thickness keratinocyte atypia and disordered maturation. Keratin pearls are present along with overlying compact hyperkeratosis and parakeratosis.

## Discussion

This case illustrates the diagnostic challenge of detecting nail unit malignancy amid diffuse cGVHD. Dystrophic nail changes can readily mask neoplastic transformation.^3^ Clinicians should maintain a low threshold for biopsy when a single nail develops new symptoms, shows asymmetric worsening, or fails to respond to standard therapy, even in the context of diffuse chronic nail disease. These focal changes may signal malignant transformation and warrant further workup.^4^ When nail unit malignancy is suspected, biopsy technique should be tailored to the anatomic localization of findings. For lesions confined to the nail bed, a longitudinal nail bed biopsy is preferred, as it allows adequate sampling while minimizing disruption to the nail matrix. When distal matrix involvement is suspected, as in this case, a longitudinal biopsy extending from the nail bed into the distal matrix can be performed following partial nail plate avulsion and reflection of the proximal nail fold to expose the matrix.^5^ Preserving the proximal nail matrix is critical, as iatrogenic injury at this site can result in permanent nail dystrophy, a particularly meaningful consideration in patients who already carry baseline nail changes from cGVHD. Use of a digital tourniquet optimizes visualization and hemostasis. Faux nail placement following avulsion may support healing and reduce postoperative discomfort. Prompt biopsy is especially important in the nail unit, where delayed diagnosis can increase morbidity and potentially necessitate more extensive treatment.

Although chronic nail cGVHD may theoretically contribute to malignant transformation through sustained inflammation, epithelial disruption, and impaired barrier function,^6^ a causal relationship cannot be established from a single case. High-risk HPV and prolonged immunosuppression remain well-supported independent risk factors for subungual SCC, and their contributions in this case cannot be excluded despite negative standard HPV testing.^3^ The histologic features suggesting possible HPV-mediated changes underscore the limitations of current testing panels in detecting low-risk or novel subtypes. This is particularly relevant in immunocompromised patients, where atypical presentations and mixed pathogenic contributors may coexist.

As BMT survivorship continues to improve, dermatologic follow-up should include careful nail examination with attention to any nail that changes differently from others. Focal symptoms such as bleeding, tenderness, or progression despite treatment should prompt timely biopsy and consideration of additional workup for malignancy. More broadly, this case highlights the need to reassess longstanding diagnoses when a focal lesion begins to diverge from the expected clinical pattern. When a single nail diverges from a background of diffuse dystrophy, whether from cGVHD, nail psoriasis, lichen planus, or other systemic conditions, prompt referral to a dermatologist specializing in nail disorders or an experienced dermatologic surgeon is warranted. Subspecialty expertise ensures accurate anatomical mapping of the lesion, appropriate biopsy technique selection, and preservation of functional nail tissue.

### Declaration of generative AI and AI-assisted technologies in the writing process

Not applicable.

## Conflicts of interest

None disclosed.
